# Protective effect of *Artemisia absinthium *on 6-hydroxydopamine-induced toxicity in SH-SY5Y cell line 

**Published:** 2021

**Authors:** Roghayeh Rashidi, Ahmad Ghorbani, Hassan Rakhshandeh, Seyed Hadi Mousavi

**Affiliations:** 1 *Pharmacological Research Center of Medicinal Plants, Mashhad University of Medical Sciences, Mashhad, Iran*; 2 *Medical Toxicology Research Center, Mashhad University of Medical Sciences, Mashhad, Iran*; 3 *Department of Pharmacology, Faculty of Medicine, Mashhad University of Medical Sciences, Mashhad, Iran*

**Keywords:** Artemisia absinthium, Parkinson's disease, ROS, 6-hydroxy dopamine

## Abstract

**Objective:**

Parkinson’s disease (PD) is a neurodegenerative disorder characterized by loss of dopaminergic neurons. Several experimental studies have shown neuroprotective and antioxidant effects for *Artemisia absinthium*. The present study was designed to assess the effect of *A. absinthium* on 6-hydroxydopamine (6-OHDA)-induced toxicity in SH-SY5Y cells.

**Materials and Methods:**

SH-SY5Y cells were treated with ethanolic extract of *A. absinthium *for 24 hr and then, exposed to 6-OHDA (250 μM) for another 24 hr. MTT (3-(4, 5-dimethylthiazol- 2-yl)-2, 5-diphenyl tetrazolium bromide) assay was used for evaluation of cell viability. Moreover, the rate of apoptosis was measured using propidium iodide (PI) staining. The amount of intracellular reactive oxygen species (ROS) and malondialdehyde (MDA) was also measured using 2’, 7’–dichlorofluorescin diacetate (DCFDA) fluorometric method. Determination of glutathione (GSH) and superoxide dismutase (SOD) activity was done by colorimetric assay using DTNB [5, 5′-Dithiobis (2-nitrobenzoic acid)] and pyrogallol respectively.

**Results:**

While 6-OHDA significantly increased ROS and apoptosis (p<0.001), the extract of *A. absinthium *significantly reduced ROS and cell apoptosis at concentrations ranging from 6.25 to 25 μg/mL (p<0.01 and p<0.001 respectively). Also, the extract significantly reduced MDA level in comparison with 6-OHDA (p<0.001). The GSH level and SOD activity were increased by the extract.

**Conclusion:**

Findings of the current study showed that *A. absinthium* exerts it effect through inhibiting oxidative stress parameters and it can be considered a promising candidate to be used in combination with the conventional medications for the treatment of neurodegenerative disorders, such as Parkinson's disease.

## Introduction

Parkinson’s disease (PD) is a common neurological disease that affects elderly patients with a prevalence of 1 to 4% (Hujoel et al., 2018 ▶). Loss of dopaminergic neurons is one of the main pathophysiologic characteristics of PD. Despite the fact that the reason for this neuronal degeneration is not completely known, various experimental studies have shown that reactive oxygen species (ROS) and oxidative pressure are involved in the loss of neurons (Zhang et al., 2000 ▶; Fiskum et al., 2003). In fact, apoptosis is one of the most important mechanisms involved in the pathogenesis of neurodegenerative disorders. Several human studies as well as *in vivo *and *in vitro *experimental findings suggested that apoptosis induces cell death in dopaminergic neurons in PD (Hartmann et al., 2001 ▶). 

6-Hydroxydopamine (6-OHDA) that is a usual neurotoxin, is widely used for induction of cell damage. This agent is used in both animals and *in vitro *studies of PD. Oxidative stress and apoptosis are involved in the toxicity of 6-OHDA (Haghdoost-Yazdi et al., 2014 ▶). In numerous studies, 6-OHDA has been used in screening of medicinal plants for management of PD (Levites et al., 2002 ▶; Chaturvedi et al., 2006 ▶; Zhang et al., 2012 ▶; Pasban-Aliabadi et al., 2013 ▶).

*Artemisia absinthium* Linn. is a medicinal plant that belongs to the family of Asteraceae (Nikhat et al., 2013 ▶). This plant is popularly known as “Wormwood” and Afsantin (Ueda and Kato., 1980 ▶). In traditional medicine, *A. absinthium *(L.) is known for its antispasmodic, stomachic, cardiac stimulant, anthelmintic, and anti-inflammatory properties, and is generally used to improve memory and mental abilities (Wake et al., 2000 ▶; Guarrera., 2005 ▶). Several experimental studies have shown neuroprotective effects for *A. absinthium*. It was reported that administration of essential oil of *A. absinthium* decreased H_2_O_2_ toxicity (Mahmoudi et al., 2009 ▶). Also, it was shown that ethyl acetate fraction of *A. absinthium* can reduce ischemia-induced oxidative stress in the brain (Bora and Sharma, 2010 ▶). To the best of our knowledge, no study has evaluated the effect of* A. absinthium* on PD. Therefore, the present study was designed to assess the effect of *A. absinthium* on 6-OHDA-induced toxicity in SH-SY5Y cells.

## Materials and Methods

Fluorescent probe 2, 7-dichlorofluorescein diacetate, 3-(4, 5-dimethylthiazol-2-yl)-2, 5-diphenyl tetrazolium (MTT), propidium iodide (PI), sodium citrate, and Triton X-100 were purchased from Sigma. DMEM and Fetal Bovine Serum (FBS) were purchased from Gibco Life Technologies (Grand Island, NY, USA). 

**Plant material and extraction**

Aerial parts of *A. absinthium *were collected in July 2016 from the mountains of Allah Akbar of Dargaz, PROVINCE, Iran. The plant was identified in the Herbarium of Khorasan Razavi Agricultural and Natural Resources Research Center. A voucher specimen (No. 11856) was deposited in the herbarium of Khorasan Razavi Agricultural and Natural Resources Research Center. To prepare the extract of* A. absinthium*, 200 g of the dried aerial parts was powdered, and the provided powder was percolated with 1500 ml of EtOH 70% for 72 hr. After filtering the extract, the solvents were allowed to evaporate at 45^°^C under reduced pressure to obtain the crude extracts.

**Standardization of the extract of** ***A. absinthium***

The hydroalcoholic extract of *A. absinthium* was standardized based on phenolic content. A sample of 20 µl of the extract (10 mg/ml) or gallic acid as standard was added to 100 µl of Folin-Ciocalteu reagent. After adding 300 µl of sodium carbonate solution (1 mol/L), the volume of the mixture was adjusted to 2 ml with deionized water. After 2 hr, the optical density was measured at 765 nm by a spectrometer. The standard curve was drawn for gallic acid (0, 50, 100, 150, 250, and 500 mg/L) and the level of phenolic compounds in the extract was expressed as milligram of gallic acid equivalents (Hosseini et al., 2017 ▶).

**Cell culture and treatment**

The SH-SY5Y (human neuroblastoma) cells were provided from Pasture Institute, Tehran, Iran. Dulbecco's Modified Eagle Medium (DMEM) supplemented with 10% v/v of FBS and 100 units/ml of penicillin/streptomycin mixture, was used for cell culture, and cells were maintained at 37°C in 5% v/v CO_2_. For experiments, cells were seeded at a density of 1×10^5^ cells in the plastic flasks. *A. absinthium* extract was dissolved in DMSO (50 mg/ml) and stored at -20°C. To study the protective effect of *A. absinthium*, cells were pretreated with the extract for 24 hr and then, incubated with 6-OHDA (250 µM) for 24 hr.

**Cell viability assay**

MTT assay was used to evaluate cell proliferation as described previously (Boncler et al., 2014 ▶). After treatment, the cells were incubated with MTT solution (0.5 mg/ml in final volume) prepared in fresh medium and added to each well. Following 4 hr incubation, the absorbance was quantified at the 570 nm using an ELISA microplate reader. 

**Assessment of ROS level**

The 2, 7′-dichlorofluorescin diacetate was used for detection of ROS level (Aranda et al., 2013 ▶). 

SH-SY5Y cells were seeded in a 96-well plate at a density of 5×10^3^ cells for 24 hr. At the end of treatment, DCFH-DA (20 μM) was added to each well and the cells were incubated for 30 min. Finally, the fluorescence intensity was read using a fluorescent microplate reader at an excitation wavelength of 485 nm and an emission wavelength of 520 nm.

**Assessment of apoptosis level**

Flow cytometry and PI staining of treated cells were performed to determine the number of apoptotic cells in the sub-G1 peak (Riccardi and Nicoletti., 2006 ▶). The cells were cultured in 12-well plates (2×10^5^ cells in well) for 1 day. After treatment, the cells were washed with phosphate-buffered saline, harvested, and incubated with 400 μL of hypotonic buffer (50 μg/ml PI in 0.1% sodium citrate and 0.1% Triton X-100) at 4°C for 30 min in the dark before flow cytometry analysis (BD Biosciences, CA, USA).

**Assessment of lipid peroxidation level**

Malondialdehyde (MDA) assay was used to estimate the level of lipid peroxidation. At the end of the incubation, the cells were scraped and lysed by homogenization in ice-cold 1.15% KCl. Then, the cells were centrifuged at 13,000 rpm at 4°C for 30 min (Zhang et al., 2017 ▶). Next, 400 μl of trichloroacetic acid (TCA) (15%) and 800 μl of thiobarbituric acid (TBA) (0.7%) were added to 500 μl of cell suspension. After vortexing the mixture, 200 μl of the sample was added to a 96-well plate. Then, the fluorescence intensity was read at excitation/emission of 530/550 nm. 

**Determination of the GSH level**

Glutathione (GSH) containing sulfhydryl group was measured through the formation of yellow color in the presence of DTNB [5, 5′-Dithiobis (2-nitrobenzoic acid)]. In this regard, cells were washed twice with phosphate-buffered saline and then lysed by 5% TCA to extract cellular GSH (Ka et al., 2003 ▶). After centrifugation at 14,000 rpm for 10 min, the denatured proteins were removed. In brief, TCA extract (500 mL) was mixed with 1 ml of a reaction mixture containing 0.1 M sodium phosphate buffer (pH 7.5), and 0.6 mM DTNB, and then, the rate of increase in absorbance was measured at 412 nm for 2 min using a spectrophotometer. 

**Determination of SOD activity**

To measure the activity of superoxide dismutase (SOD), a 6-well plate was used. The cells were incubated with effective concentrations of extract for 24 hr, and then incubated with 250 μM 6-OHDA for 24 hr. SOD activity was determined by spectrophotometry (at 405 nm) based on inhibition of pyrogallol autoxidation as described previously (Keshavarz et al., 2017 ▶). The rate of pyrogallol autoxidation in tris-cacodylic acid buffer (0.05 M, pH 8.2) was determined (A_1_). The autoxidation of pyrogallol was evaluated under the same conditions after addition of 20 µl of sample (A_2_). The inhibition percentage of pyrogallol oxidation was determined using the following formula:

% Inhibition: (A_1_-A_2_/A_1_)×100

**Statistics analysis**

For determination of differences among groups, one-way ANOVA followed by the Tukey-Kramer *post hoc* test was used. All results are presented as mean±SD and p-values below <0.05 were regarded as statistically significant. Each experiment was repeated at least three times.

## Results

**Phenolic content of** ***A. absinthium***

The content of total phenols in the hydroalcoholic extract of *A. absinthium* was 152 mg gallic acid equivalent per gram of the crude extract.

**Effects of** ***A. absinthium*** **on neurotoxicity induced by 6-OHDA**

**Figure 1:**
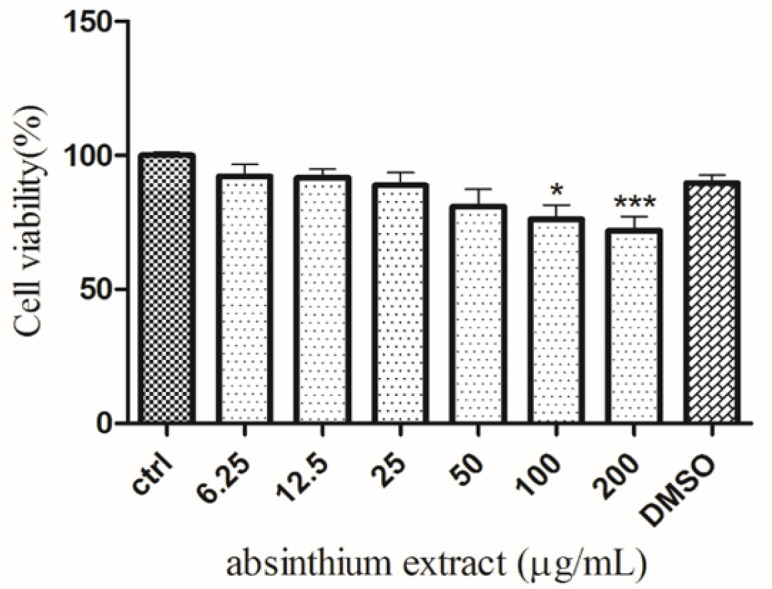
Effects of *A. absinthium *extract on cell viability. The viability of SH-SY5Y cells was determined by MTT assay after treatment with *A. absinthium *extract (6.25 to 200 μg/ml) for 48 hr. The data is presented as the mean±SD of three independent experiments. ^*^p<0.05 and ^***^p<0.001 compared to the control group

**Figure 2:**
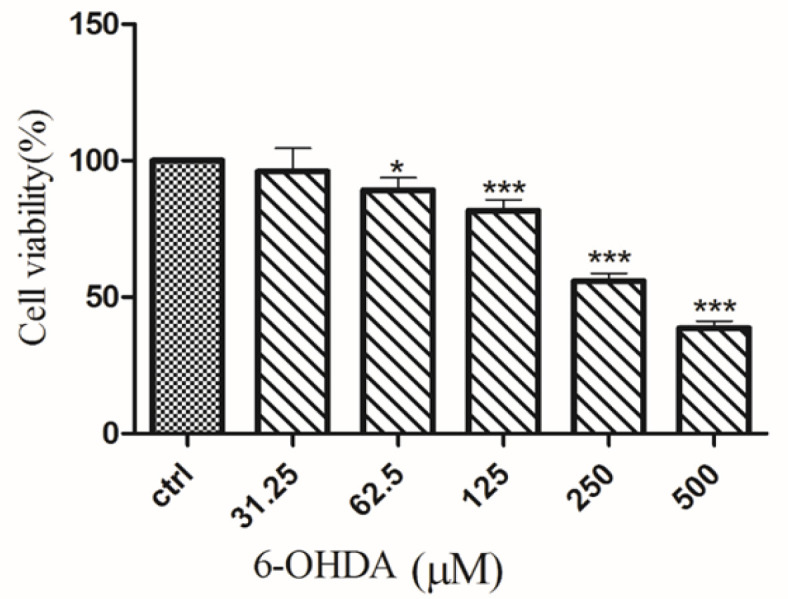
Effects of 6-OHDA on cell viability. The viability of SH-SY5Y cells was determined by MTT assay after treatment with 6-OHDA (31.25 to 500 μM) for 24 hr. The data is presented as the mean±SD of three independent experiments. ^*^p<0.05 and ^***^p<0.001 compared to the control group

**Figure 3:**
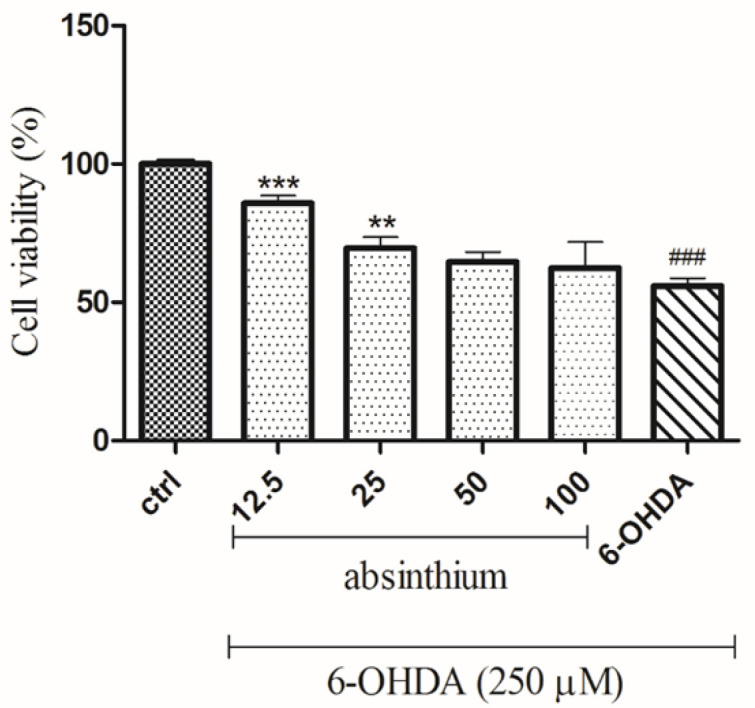
Effects of *A. absinthium *extract on 6-OHDA (250)-induced SH-SY5Y cell viability. SH-SY5Y cells were pretreated with* A. absinthium *extract (12.5 to 100 μg/mL) for 24 hr then exposed to 6-OHDA (250 μM) for 24 hr, and cellular viability was assessed by MTT assay. The data is presented as the mean±SD of three independent experiments. **p< 0.01 and ***p<0.001 compared with the 6-OHDA group, ### p<0.001 compared with the control group

Before evaluating the neuroprotective effect of *A. absinthium*, its possible effects on cell viability were tested. None of the concentrations of the three extracts tested decreased the viability of SH-SY5Y cells after 24 hr (Figure 1). 6-OHDA (250 μM) significantly decreased cell viability by 50% compared to untreated cells (p<0.001; Figure 2). *A. absinthium* extract (12.5 and 25 μg/ml) significantly inhibited 6-OHDA-induced cell toxicity (p<0.001, and p<0.01, respectively; Figure 3).

**Effects of** ***A. absinthium*** **on the level of ROS**

**Figure 4:**
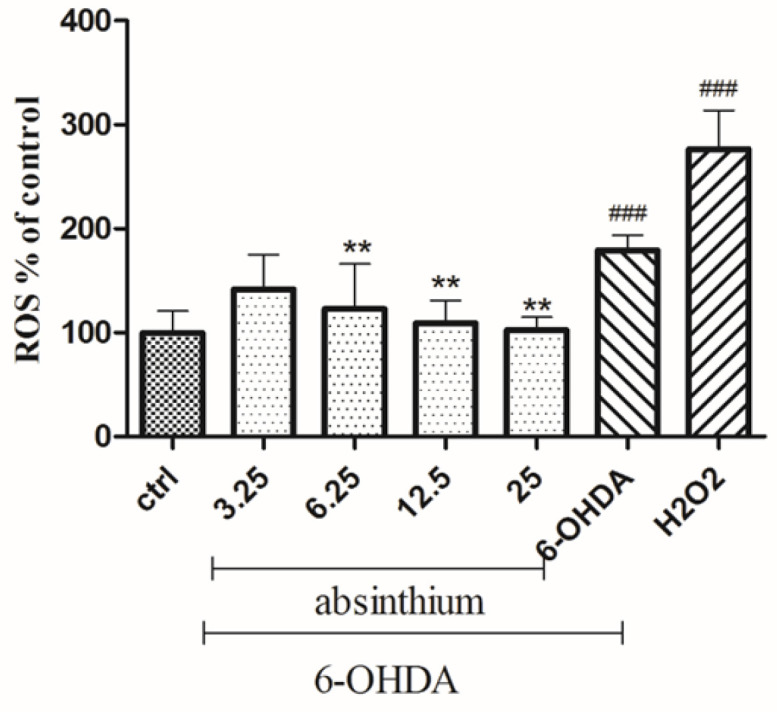
Effects of *A. absinthium *extract on 6-OHDA (250 μM)-induced ROS production. SH-SY5Y cells pretreated with *A. absinthium *extract (3.25 to 25 μg/ml) for 24 hr, and then exposed to 6-OHDA (250 μM) for 24. The data is presented as the mean±SD of three independent experiments. **p<0.01 compared with the 6-OHDA group, ###p<0.001 compared with the control group

Intracellular level of ROS significantly increased in the cells cultured in the presence of 6-OHDA. Pre-treatment with *A. absinthium* extract (12.5 and 25 μg/ml) significantly suppressed the increased ROS generation compared to untreated cells (p<0.01; Figure 4). 

**Effects of** ***A. absinthium*** **on 6-OHDA-induced apoptosis**

**Figure 5:**
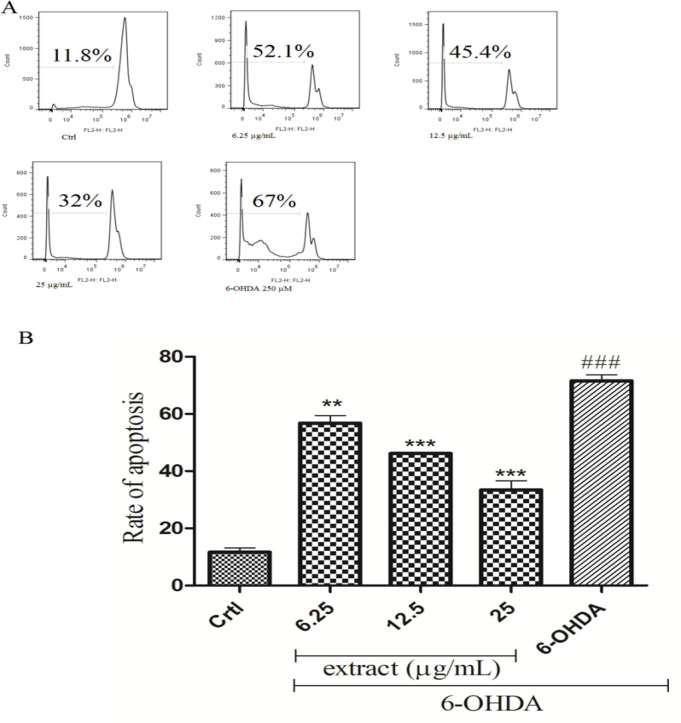
The effects of *A. absinthium *extract on DNA fragmentation induced by 6-OHDA in SH-SY5Y cells. Flow cytometry analysis of DNA fragmentation was performed with PI method. A: Flow cytometry histograms of different groups and B: column bar graph of percentage of cells with DNA fragmentation. Data is expressed as the mean±SD of three separate experiments. **p<0.01 and ***p<0.001 compared with the 6-OHDA group, ###p<0.001 compared with the control group

Incubation of SH-SY5Y cells with 6-OHDA significantly (p<0.001) increased the percentage of apoptotic cells compared to control cells (67% and 11.8%, respectively). Pre-treatment with *A. absinthium* extract (6.25 and 25 μg/ml) significantly decreased 6-OHDA-induced apoptosis (p<0.01, and p<0.001 respectively) (Figure 5).

**Effect of** ***A. absinthium*** **on lipid peroxidation**

**Figure 6:**
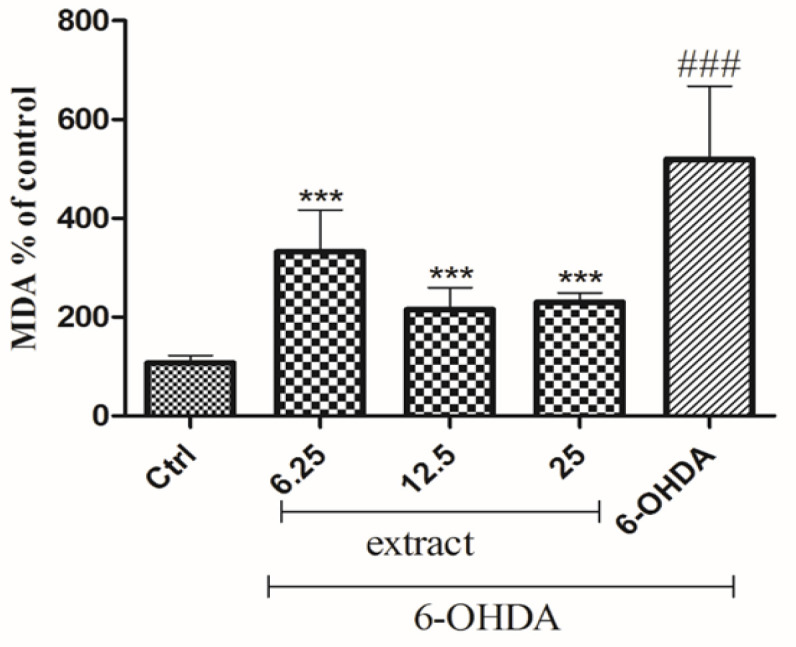
The effects of *A. absinthium *extract on MDA content under 6-OHDA treatment in SH-SY5Y cells. The cells were pretreated with different concentrations of the extract (6.25 to 25 µg/ml) for 24 hr, then exposed to 250 µM 6-OHDA and incubated for 24 hr. Results are the means±SD from three independent experiments.^ ***^p<0.001 compared with the 6-OHDA group. ^###^p<0.001 compared with the control group

As shown in Figure 6, incubation of the cells with 6-OHDA significantly increased MDA level (356%, p<0.001) as compared to the control group. The content of MDA was significantly decreased in the cells pre-treated with 6.25 µg/mL (332.502%, p<0.01), 12.5 µg/ml (215.151%, p<0.01) and 25 µg/ml (195%, p<0.001) of *A. absinthium* extract, compared to 6-OHDA group (Figure 6).

**Effect of** ***A. absinthium*** **on 6-OHDA-decreased GSH level**

**Figure 7:**
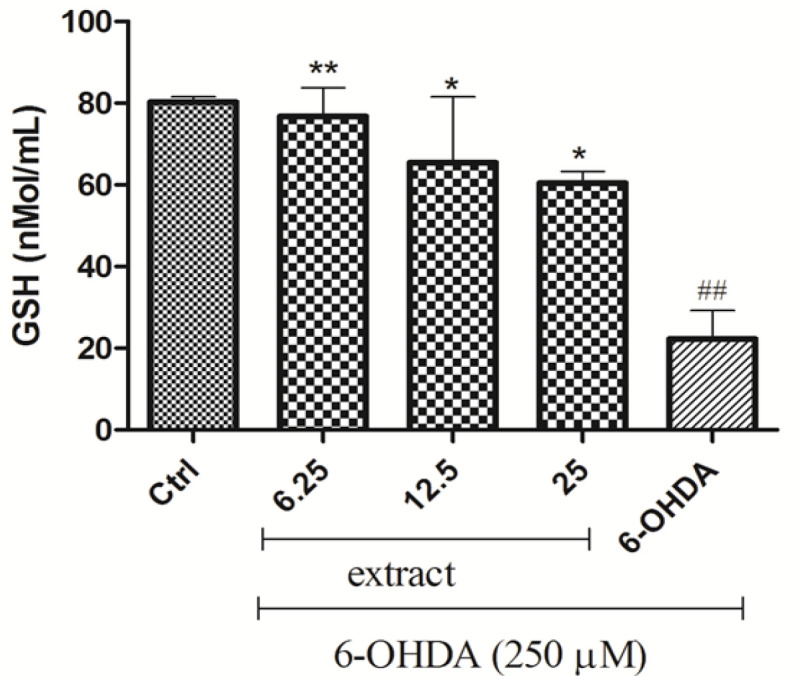
Effect of *A. absinthium *on 6-OHDA-induced GSH reduction. Cells were treated with different concentrations of the extract (6.25, 12.5 and 25 µg/ml) before exposure to 250 mM of 6-OHDA. Results are the means±SD from three independent experiments. *p<0.05 and ^**^p<0.01 compared with the 6-OHDA group. ^##^p<0.01 compared with the control group

6-OHDA (250 µM) significantly decreased GSH level in comparison with the control group (p<0.01). Pre-treatment of cells with 6.25, 12.5 and 25 µg/mL of *A. absinthium* extract, significantly increased GSH (p<0.01, p<0.05 and p<0.05, respectively) (Figure 7). 

**Effect of** ***A. absinthium*** **on 6-OHDA-decreased SOD activity**

**Figure 8:**
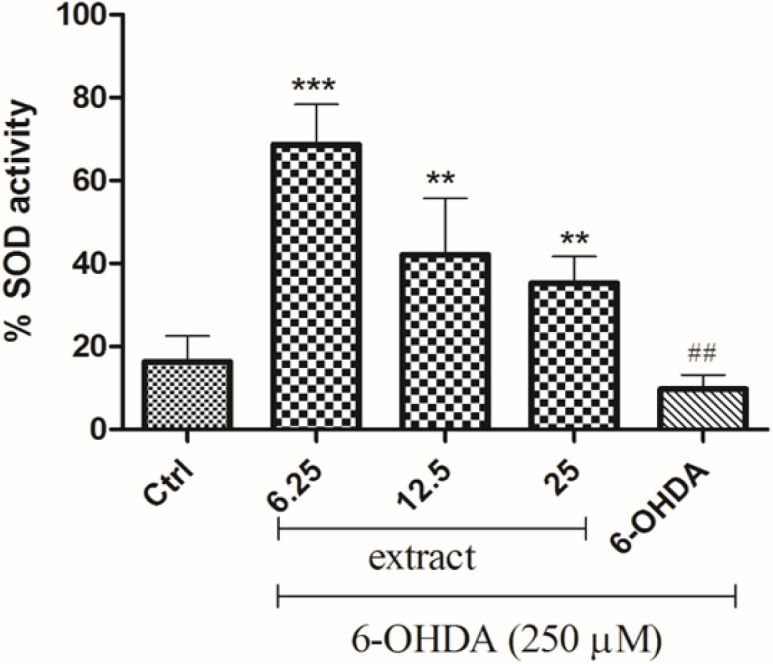
Effect of *A. absinthium* extract on the 6-OHDA-induced activation of SOD in SH-SY5Y cells. Cells were pretreated with 6.25 to 25 μg/ml of extract for 24 hr, and then incubated in the presence of 250 μM 6-OHDA for 24 hr. Protein was extracted and the activity of SOD was detected. Results are the means±SD from three independent experiments. ^**^p<0.01 and ***p<0.001 compared with the 6-OHDA group. ^##^p<0.01 compared with the control group

As shown in Figure 8, 6-OHDA reduced SOD activity compared to the control group. Pre-treatment of SH-SY5Y cells with extract (6.25 to 25 µg/mL) for 24 hr significantly increased SOD activity in the cells treated with 6-OHDA.

## Discussion

Here, we examined the possible protective effect of *A. absinthium *against 6-OHDA neurotoxicity. Extreme production of ROS increase the lipids, proteins and DNA oxidation products, which in turn, cause cellular damage and subsequent cell death. Also, malondialdehyde (MDA) and increased lipid peroxidation are produced by ROS (Bora and Sharma., 2011 ▶). Accordingly, MDA reduction was investigated in the present study. Recent studies have shown that reduction in the level of ROS plays an important role in the protection against neurodegenerative diseases such as PD (Lin et al., 2015 ▶). Also, *A. absinthium* extracts can be used as a neuroprotective agent against diseases associated with oxidative stress. Our data showed that pre-treatment of SH-SY5Y cells with *A. absinthium* extract (12.5 and 25 μg/ml) increased the cell viability and decreased the level of ROS, MDA, and apoptosis. 

Previous studies have reported a number of neuroprotective effects for *A. absinthium*. For example, the methanolic extract of* A. absinthium* (100 and 200 mg/kg) inhibited the brain oxidative stress and damage created by middle cerebral artery occlusion in rats (Bora and Sharma., 2010 ▶). The aqueous *A. absinthium* extract (200 mg/kg) also reduced the neurotoxicological damage induced by lead in rats (Kharoubi et al., 2011 ▶). In another study, it was shown that caruifolin D derived from *A. absinthium* significantly inhibited lipopolysaccharide-stimulated ROS production in BV-2 cells (Zeng et al., 2015 ▶). Also, *A. absinthium *extract (IC_50 _concentration of less than 1 mg/ml) showed nicotinic and muscarinic receptor activity in homogenates of human cerebral cortical membrane (Wake et al., 2000 ▶). Li and Ohizumi (2004) ▶ reported that the methanolic extract of *A. absinthium *upgraded neurite outgrowth instigated by nerve growth factor in PC12 cells (Li and Ohizumi., 2004 ▶).

Bora and Sharma (2011) ▶ previously reported that* A. absinthium* methanolic extract (400 mg/kg, i.p.) possesses potent antioxidant properties in mice (Bora and Sharma., 2011 ▶). Amat and his colleagues (2010) ▶ reported that the aqueous extract of *A. absinthium* increased the antioxidant enzymes such as, SOD and GPx and reduced the MDA level in the liver tissue (Amat et al., 2010 ▶). In another study, hydroxyflavone (p7F) derived from *A. absinthium* at the concentrations of 25 to 100 µg/mL inhibited the cytotoxicity of H_2_O_2_-induced ROS in RAW 264.7 cells (Lee et al., 2004 ▶). It has been reported that the antioxidant activity of *A. absinthium* increased depending on the type and concentration of the applied plant extracts in the following order ethyl acetate>methanol>nbutanol>chloroform>petroleum ether> remaining water extracts (Canadanovic‐Brunet et al., 2005 ▶).

The results of this study indicated that *A. absinthium* etanolic extract has neuroprotective effects against 6-OHDA-induced oxidative SH-SY5Y cells death through reduction of MDA, ROS and apoptosis. The present investigation indicated a novel therapeutic potential of *A. absinthium *for protection of SH-SY5Y cells against 6-OHDA-induced toxicity. Findings have shown that this plant may be used for treatment of neurodegenerative diseases such as PD, but elucidating the underlying mechanisms of this protection needs further *in vitro* and *in vivo* investigations.
